# Using a Riboswitch Sensor to Detect Co^2+^/Ni^2+^ Transport in *E. coli*

**DOI:** 10.3389/fchem.2021.631909

**Published:** 2021-02-15

**Authors:** Xiaoying Wang, Wei Wei, Jing Zhao

**Affiliations:** ^1^ State Key Laboratory of Coordination Chemistry, Chemistry and Biomedicine Innovation Center (ChemBIC), School of Chemistry and Chemical Engineering, Nanjing University, Nanjing, China; ^2^ School of Life Sciences, Nanjing University, Nanjing, China

**Keywords:** Co^2+^/Ni^2+^ detection, CoNi riboswitch, fluorescent protein, whole-cell sensor, metal ion transport

## Abstract

Intracellular concentrations of essential mental ions must be tightly maintained to avoid metal deprivation and toxicity. However, their levels in cells are still difficult to monitor. In this report, the combination of a Co^2+^Ni^2+^-specific riboswitch and an engineered downstream mCherry fluorescent protein allowed a highly sensitive and selective whole-cell Co^2+^/Ni^2+^ detection process. The sensors were applied to examine the resistance system of Co^2+^/Ni^2+^ *in E. coli*, and the sensors were able to monitor the effects of genetic deletions. These results indicate that riboswitch-based sensors can be employed in the study of related cellular processes.

## Introduction

Nickel and cobalt are employed by considerable enzymes in germ, fungus, flora, and fauna as critical cofactors (Kobayashi and Shimizu, 1999; Watt and Ludden, 1999). Co^2+^/Ni^2+^ is usually found and reconciled in proteins and cobalamin (e.g., vitamin B12) (Li and Zamble, 2009, Zhang et al., 2009; Okamoto and Eltis, 2011; Cheng et al., 2016). Transition metal in excess could be toxic in cells; the quantity equilibrium of the metal ions in cells impacts life models of organic lives critically. For this reason, to keep balance of metal ions, special resistance systems were formed by microorganism to confine the quantity of metal ions to the optimal scope. (Okamoto and Eltis, 2011; Higgins et al., 2012)

In spite of the importance of Co^2+^/Ni^2+^ for the functions inside the cells, it is still very difficult to test the concentration of Co^2+^/Ni^2+^ inside the cells. Thus far, various chemical probes have been developed to detect the concentration of Co^2+^/Ni^2+^, whereas the experiments based on the mentioned techniques were mostly performed outside of the living body by employing expensive apparatuses and following intricate steps (Zhu H et al., 2015). Recently, a scheme for testing Co^2+^/Ni^2+^ as assisted by a whole-cell biosensor based on a metal regulatory protein, which is relatively few, has been proposed (Tibazarwa et al., 2001; Duprey et al., 2014). In comparison with the chemical probes, the biological ones have the advantages in steps, which are more directed. Furthermore, the operations of whole-cell biosensors are more specific and biocompatible.

The whole-cell sensors are living microorganisms that produce a specific, qualitative, or quantitative output in response to substance of interest (Bereza-Malcolm et al., 2015). The sensor reconciles a molecular import region to sense metal ions and an export region to detect signals; briefly, the metal ions included in sensing elements motivate an export signal able to be detected, for instance, colorimetric, fluorescent, or luminescent proteins (Verma and Singh, 2005; Hynninen and Virta, 2010; Park et al., 2013). The import region generally refers to the genetics of metallic balance rules inside microorganisms. The sensor combining the gold-particular prober protein GolS by *Samonella* gol regulon and engineered downstream red fluorescent protein is an instance of the heavy metal whole-cell biosensor to test gold ion (Wei et al., 2012).

Inartificial metal regulatory RNA, as well as metal regulation protein, has been developed to detect the metal ions (Cromie et al., 2006; Furukawa et al., 2015; Dambach et al., 2015). The name of the natural RNA is “riboswitch,” consisting of noncoding RNA regions; this RNA has the ability to selectively bind metabolites and to conduct the basic metallic processes of many organic creatures; the majority of the riboswitch have been recognized existing in the 5′ regions (5′-UTRs) of the mRNA, which have not been translated. Its function is to regularize genes at downstream in transcription or translation by structural changing (Mandal and Breaker, 2004; Breaker, 2011; Breaker, 2012). In fact, the spontaneously occurring riboswitch is suitable for substance response and backward conduction as import regions of whole-cell sensors. Moreover, coenzyme B12 riboswitch has been adopted to fabricate whole-cell biosensors to investigate the synthesis and transportation of coenzyme B12 in *E. coli* and to detect concentrations of coenzyme B12 in fermented food (Fowler et al., 2010; Fowler et al., 2013; Zhu et al., 2015). Compared with sensors based on the metal regulation protein, the riboswitch-based sensor is more responsive and sensitive to changes of metabolites within the cells.

**FIGURE 1:**
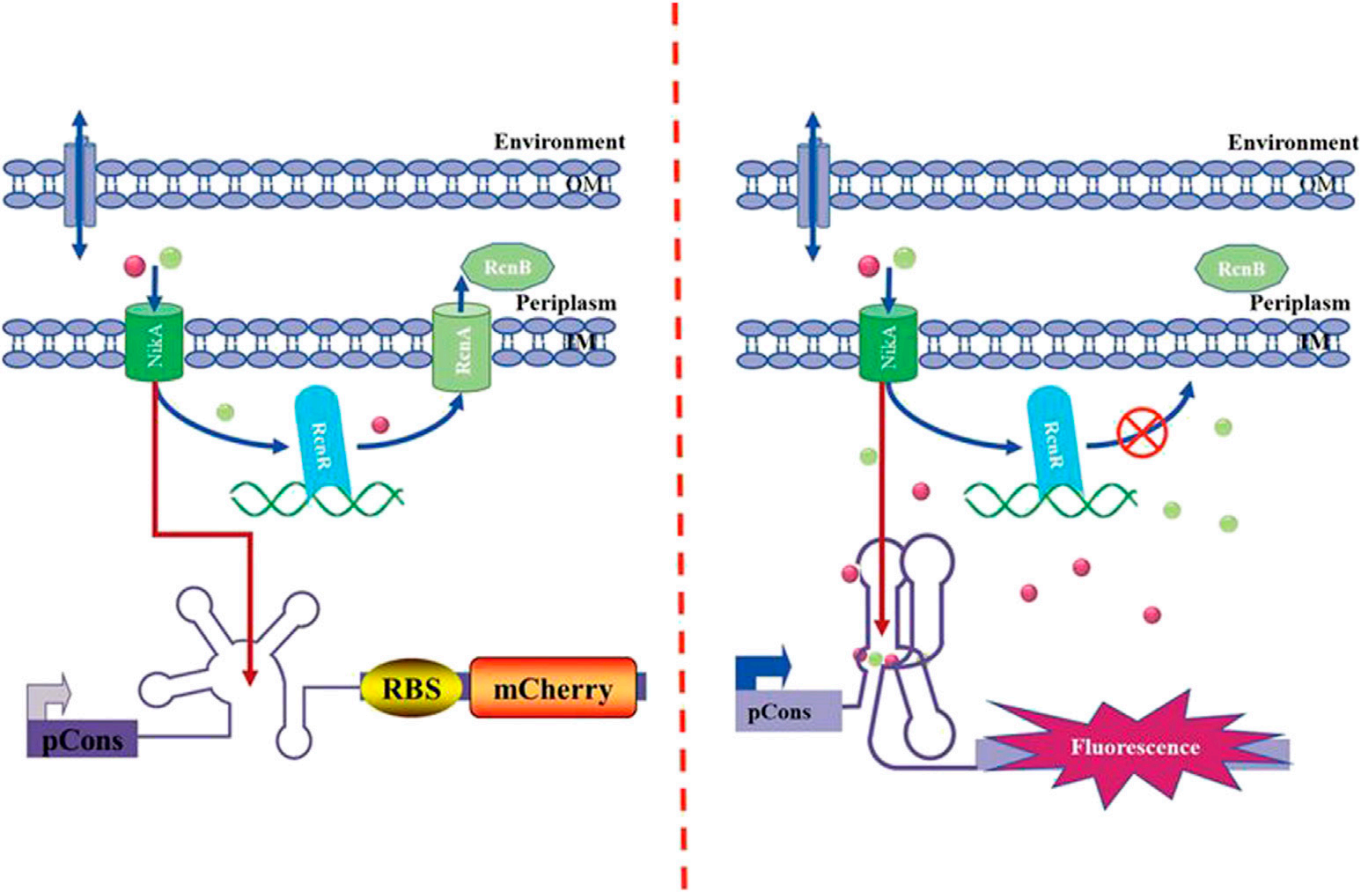
Mechanism of the NiCo riboswitch–based whole-cell biosensor.

A new conserved riboswitch with low-abundant metal ions (Co^2+^/Ni^2+^, Mn^2+^, Fe^2+^ Mg^2+^, et. al) were confirmed (Furukawa et al., 2015; Bandyopadhyay et al., 2020; Cromie et al., 2006; Price et al. 2015). The NiCo riboswitch is capable of selectively and tightly binding to micromolar Ni^2+^ and Co^2+^ (Furukawa et al., 2015). Based on the identification of the novel NiCo riboswitch, NiCo riboswitch regulating the expression of *mCherry* gene was employed to analyze Ni^2+^/Co^2+^ transport in *E. coli*. As it can be seen in Figure 1, the NiCo riboswitch prevents formation of an overlapping intrinsic terminator stem and promotes production of full-length transcription product of *mCherry* when Ni^2+^/Co^2+^ is bound; this allows for efficient transcription of the reporter gene and results in a high level of fluorescence signal output. The NiCo riboswitch selectively recognizes Ni^2+^/Co^2+^; binding with positive cooperativity, it suggests that the genes controlled by riboswitches are likely to be induced by even small changes in the cellular concentrations of Ni^2+^/Co^2+^ (Furukawa et al., 2015); this property will be useful in monitoring the changes of intracellular concentration of Ni^2+^/Co^2+^ when investigating the transport mechanism using genetic deletions.

The system of metallic resistance generally refers to the equilibrium of metal absorption and the exchange with procedure of efflux/storage. The effusion mechanism to impel cations from the inside to the outside of the cytoplasm is the major process. The Ni^2+^/Co^2+^ resistance mechanism is utilized by germs to keep the balance of metal ions (Rodrigue et al., 2005; Iwig et al., 2008; Blaha et al., 2011; Bleriot et al., 2011). The sensors applied in this study represent valuable tools for the study of these processes as they allow for sensitive detection of cellular Ni^2+^/Co^2+^ levels.

## Materials and Methods

### Plasmids and Bacterial Strains

All plasmids used in the experimental design were derived from the BioBrick Vector pSB1A2/pSB1C3 and modified as described below. Based on *E. coli* DH5α cells, the authors conducted molecular cloning. The single-gene deletion strains were *E. coli* K12 BW25113. All plasmids used in the single-gene deletion were derived from pKD46 and pUC57.

### Three Parallel CoNi Riboswitch Sensors’ Construction and Validation

Details of recombinant plasmid construction are provided in Supplementary Figure S1, and three different CoNi riboswitch sequences are provided in Supplementary Table S1. Three predicted CoNi riboswitches used in plasmid construction were reported by Breaker (Furukawa et al., 2015). By complying with one previous protocol, the gene encoding mCherry and the BioBrick Vector pSB1A2 received the construction (Wei et al., 2014). In brief, all NiCo riboswitch fragments were synthesized by GenScript. All fragments were obtained with PCR from a suitable templates and cloned into pSB1A2 by Biobrick based on restriction digestion and ligation.

### Comparison of Parallel CoNi Riboswtich Sensor Performance

The sensor plasmid was transformed into the *E. coli* strain K12 by electroporation and plated on LB agar plates containing 50 mg/ml ampicillin. Individual colonies were picked and grown overnight. Saturated cultures were transferred to fresh LB (1/100 dilution) until OD600 ≈ 0.6. Subsequently, cells were induced by the indicated metal ions for 2–4 h.

For the concentration sensitivity measurements, gradient concentrations of CoCl_2_ were 0, 50, 200, 500, 1,000 μM and gradient concentrations of NiCl_2_ were 0, 750, 1,000, 1,500, 2,000 μM.

For the selectivity measurements, different metal chlorides (Co^2+^, Ni^2+^, Ca^2+^, Cd^2+^, Cu^2+^, Cd^2+^, Cr^2+^, Fe^2+^, Fe^3+^, Li^+^, Mg^2+^, Mn^2+^, and Zn^2+^) were tested with final concentration of 100 and 1,000 μM, respectively. Cells were induced by the indicated metal ions for 1 and 4 h.

Subsequently, cells received the assay by complying with the procedures described below. 1 ml of cells was pelleted and resuspended in 1 ml of phosphate-buffered saline (PBS). For the respective biological replicate, 200 μL aliquots of the respective sample were used in triplicate into a 96-well flat bottom black plates (Corning), and fluorescence was read at 587/610 nm excitation/emission with a Safire fluorometer (Tecan). Measurements based on the three wells were averaged to determine the value for a given biological replicate. All fluorescence intensities (FIs) were normalized for cell density based on OD_600_ measurements taken with an automatic microplate reader (Tecan). All the presented FI/OD data represent the average of at least three biological replicates, as confirmed by performing at least two independent experiments.

### Optimal CoNi Riboswtich Sensor Performance Characterization and Fluorescence Imaging

For the time gradient measurements, cells were induced by 100 μM Co^2+^ for 0–10 h. For the selectivity measurements, different metal chlorides (Co^2+^, Ni^2+^, Ca^2+^, Cd^2+^, Cu^2+^, Cd^2+^, Cr^2+^, Fe^2+^, Fe^3+^, Li^+^, Mg^2+^, Mn^2+^, and Zn^2+^) were tested with final concentration of 100 and 1,000 μM. Cells were induced by the indicated metal ions for 4 h.

For the concentration sensitivity measurements, gradient concentrations of CoCl_2_ were 0–250 μM and gradient concentrations of NiCl_2_ were 0–2,000 μM.

Afterward, the images of cells were captured after the 4 h treatment with 0 and 200 μM CoCl_2_ under the fluorescence microscope (Leica, Germany) via a ×100 oil objective. Plasmid pSB1C3-pCons-RBS-EGFP-Ter (laboratory conservation) which expressed green fluorescent protein constantly acted as the control in fluorescence imaging and fluorescence detection.

### Construction of Mutant Strain

Ni^2+^/Co^2+^ resistance–related genes (*rcnA*, *rcnB*, *rcnR*, *nikA*, and *nikR*) were deleted by inserting Kan^r^ cassettes based on the λRed recombinase system following a previously published protocol (Datsenko and Wanner 2000). Single-gene deletions are elucidated in Supplementary Material. In brief, all target fragments and *kanamycin* gene were obtained using PCR from suitable templates; different genes were disrupted by direct transformation Red helper plasmid pKD46 with PCR products having short homology extensions for the targeted locus; target gene was knocked out, while Kan^r^ was knocked in. Positive mutant strains (K12ΔRcnA, K12ΔRcnB, K12ΔRcnR, K12ΔNikA, and K12ΔNikR) are confirmed by performing colony PCR.

### Application of CoNi Riboswtich Sensor in Mutant Strain

By adopting wild-type K12 as the control, this study investigated the application of the probe in the knockout strains. Electroporation-competent cells of five mutant strains containing the NiCo riboswtich sensor were prepared. For the effects of *rcnA*, *rcnB*, and *rcnR* deletion on intracellular levels of Co^2+^, cells were induced by 100 and 500 μM CoCl_2_ for 2 h. For the effects of *rcnA*, r*cnB*, *rcnR*, *nikA*, and *nikR* deletion on intracellular levels of Ni^2+^, cells were induced by 1,000 and 1,500 μM NiCl_2_ for 2 h. Cells were then assayed by employing the described procedures. The accumulation of metal ions in cells can be proved indirectly by calculating the ratio of change in fluorescence intensity relative to the blank control. To verify the correctness of the test, ICP-MS (Jiangsu Zhongpu Testing Co., Ltd) was adopted to determine the corresponding metal ion concentration.

$$\text{Change Ratio}=\frac{\left(F-F_{0}\right)}{F_{0}}\times 100\%.$$

## Results and Discussion

### Riboswtich Sensor Construction and Examination

**FIGURE 2:**
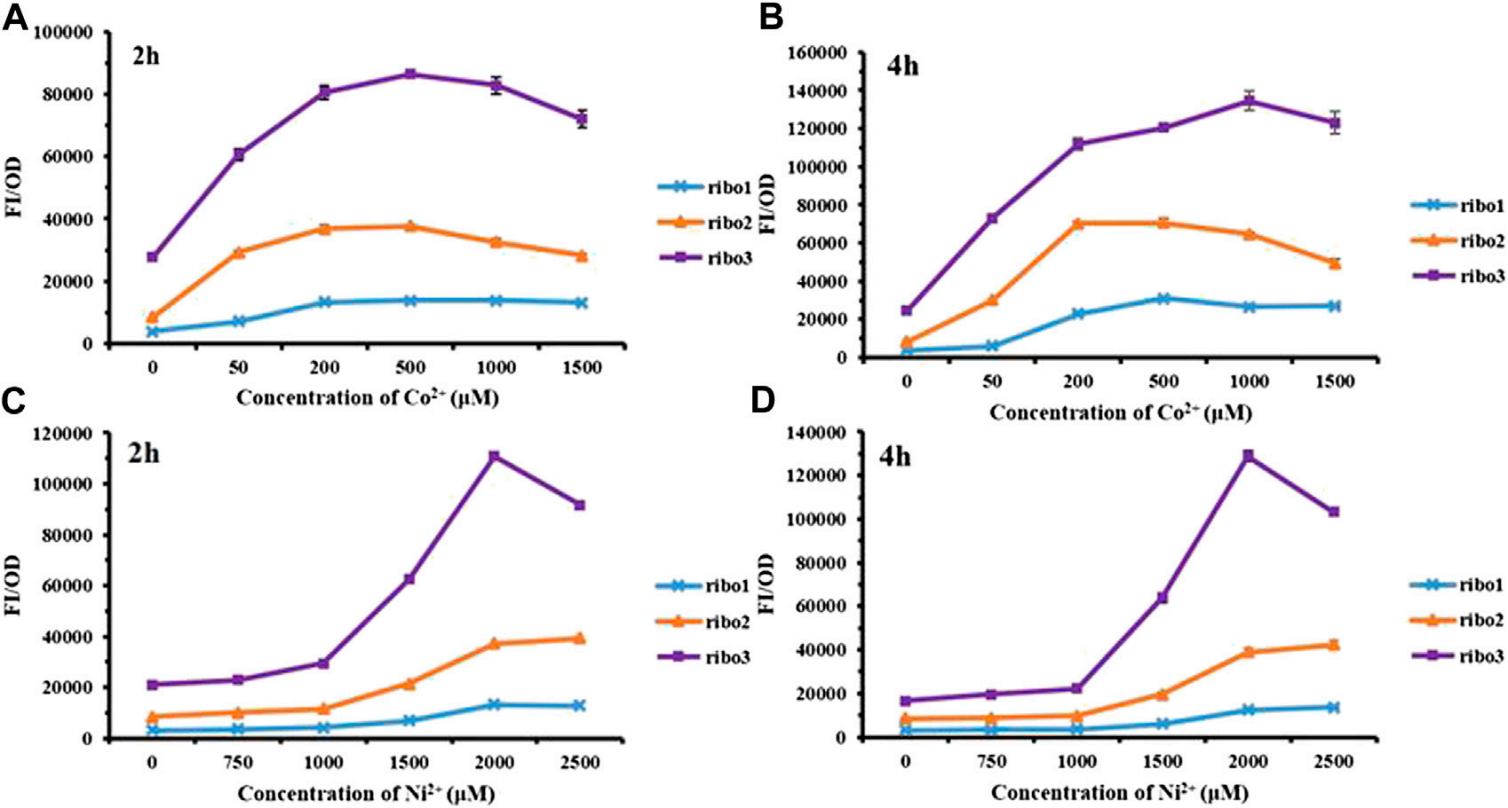
Fluorescent measurement of three parallel NiCo riboswtich sensors with concentration gradient: **(A)** FI/OD values were detected by 0–1,500 μM CoCl_2_ for 2 h. **(B)** FI/OD values were detected by 0–1,500 μM CoCl_2_ for 4 h. **(C)** FI/OD values were detected by 0–2,500 μM NiCl_2_ for 2 h. **(D)** FI/OD values were detected by 0–2,500 μM NiCl_2_ for 4 h.

The CoNi riboswitch refers to a recently identified cobalt or nickel conserved riboswitch class capable of effectively differentiating cobalt or nickel from the other metal ions (Furukawa et al., 2015). Inspired by the positive cooperativity recognition of the CoNi riboswitch, three parallel versions named Ribo1, Ribo2, and Ribo3 were constructed. The parallel probes were preliminarily measured for performance, and the results are presented in Figure 2. Overall, in the experimental concentration range, all of them responded to Co^2+^/Ni^2+^, and the fluorescence intensity increased with the concentration. According to Figures 2A and 2B, the fluorescence intensity of Co^2+^ decreased at the concentration over 1,000 μM. In addition, Figures 2C and 2D show that the fluorescence intensity of Ni^2+^ decreased at the concentration over 2,000 μM because high concentrations of Co^2+^/Ni^2+^ were more toxic to microorganisms. For time gradient, 2 h after the addition of the metal ions, the probes effectively responded to the metal ions. The probes remained active over time. The point of difference was the fluorescence background in which *E. coli* without metal ions induction, Ribo2, acted as the most suitable sensor with moderate fluorescence background.

**FIGURE 3:**
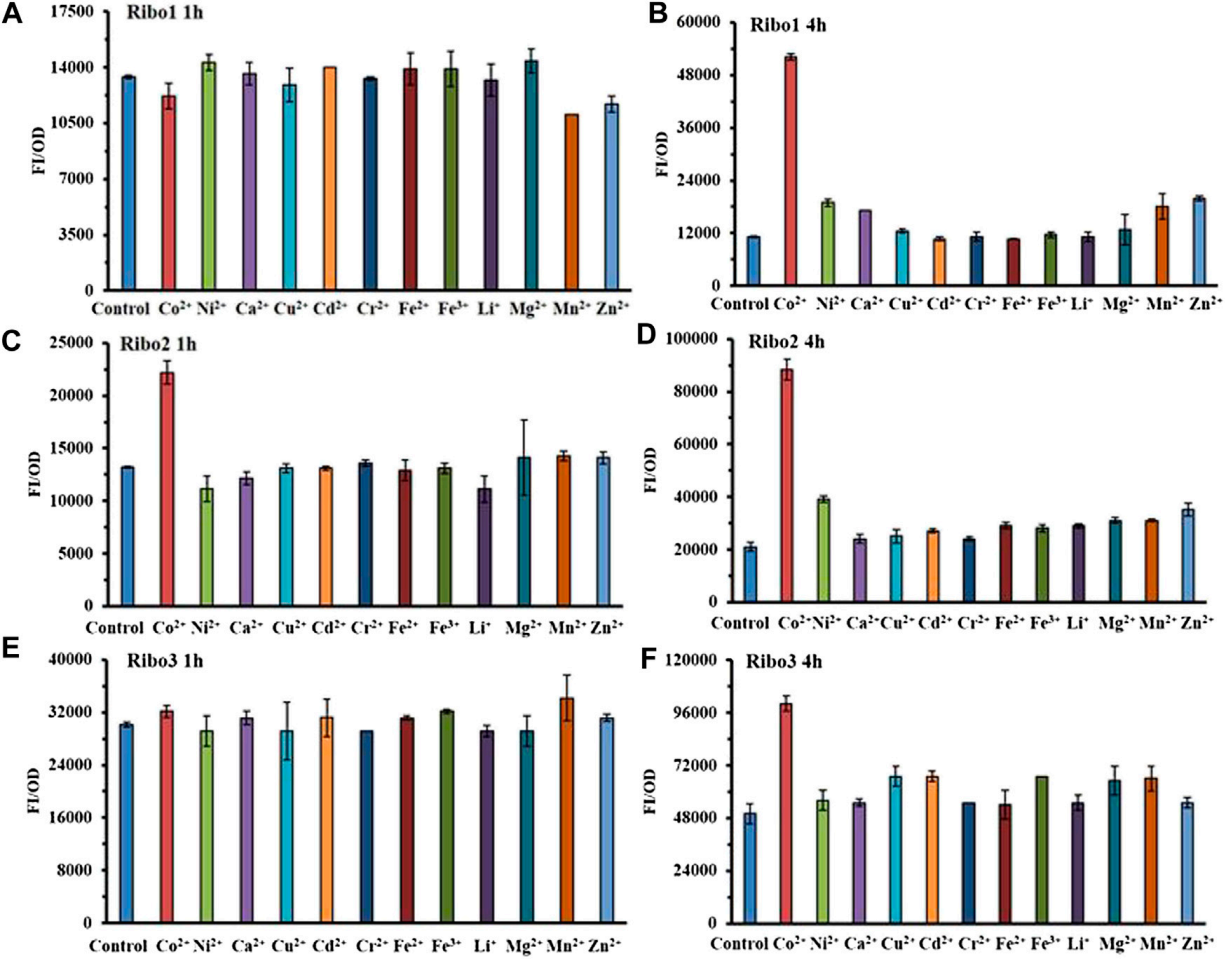
Comparison of selectivity of three parallel NiCo riboswtich sensors: **(A)**, **(B)** FI/OD values of Ribo1 were detected by 100 μM different metal chlorides for 1 h and 4 h. **(C)**, **(D)** FI/OD values of Ribo2 were detected by 100 μM different metal chlorides for 1 h and 4 h **(C)**, **(D)** FI/OD values of Ribo3 were detected by 100 μM different metal chlorides for 1 h and 4 h.

**FIGURE 4:**
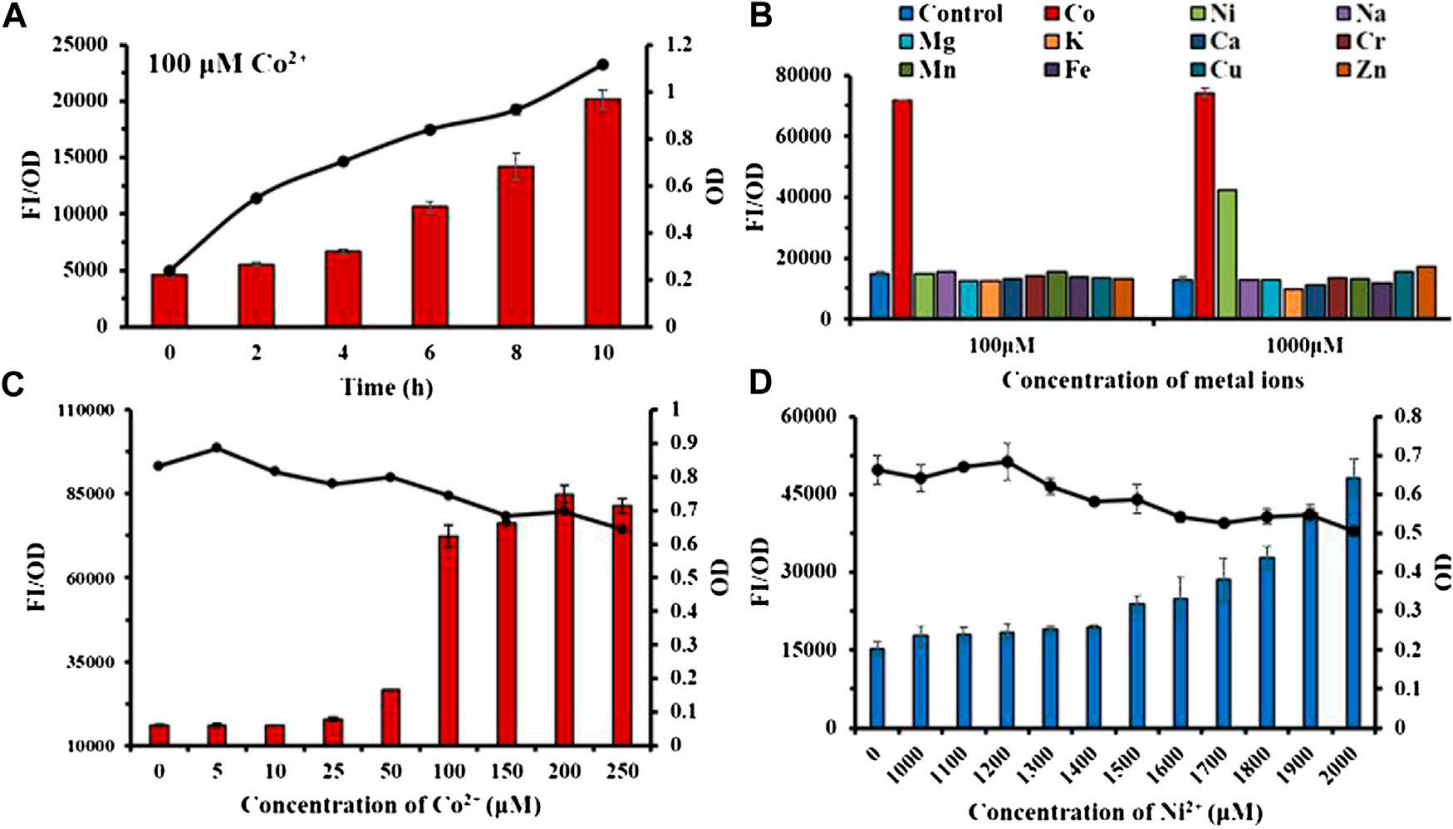
Characterization study of sensor Ribo2: **(A)**: Time gradient. **(B)** Selectivity of 100 μM and 1,000 μM. **(C)** Concentration gradient of Co^2+^. **(D)** Concentration gradient of Ni^2+^. Line graph represents the OD value.

The results of selectivity are illustrated in Figure 3, all of the three probes specifically detected Co^2+^ at the concentration of 100 μM. As indicated by Figures 3B, 3D, and 3F, 4 h data revealed all of the three probes to be very specific for Co^2+^, strongly discriminating against a range of other mental ions. According to Figures 3A, 3C, and 3D, 1 h data showed that only Ribo2 had selectivity. Given the fluorescence background and time efficient, the characteristics of probe Ribo2 were elucidated.

According to Figure 4A, the fluorescence intensity FI/OD and absorbance OD_600_ of the Ribo2 probe were continuously enhanced within 0–10 h when the Co^2+^ concentration was fixed. It responded to Co^2+^ at the concentration of 100 μM, and responded to Co^2+^ and Ni^2+^ at the concentration of 1,000 μM. In the experiment of concentration gradient, the Ribo2 probe exhibited different concentration ranges for response to Co^2+^ and Ni^2+^. At the Co^2+^ concentration gradient of 5–250 μM, the fluorescence intensity was elevated with concentration, while in 50–100μM, the fluorescence intensity displayed a rapid increase, and the OD value decreased with the increase in concentration. Under the Ni^2+^ concentration gradient of 1,000–2,000 μM, the fluorescence intensity increased with concentration, and the OD value decreased with the increase in concentration. The results showed that the Ribo2 probe could be used as a specific Co^2+^ probe in the concentration ranges of 5–250 μM. The efficient and specific probe could be used to detect intracellular ion concentration. The Ribo2 probe was selective to Ni^2+^ within 1,000–2,000 μM, and the probe could be used as a specific Ni^2+^ probe under the concentration over 1,500 μM as impacted by the difference of *E. coli* in tolerance to Co^2+^ and Ni^2+^. This roughly complies with estimated *K*_D_ values and Hill coefficient; Ribo2 RNA displays selective, tight, and cooperative binding for Ni^2+^ and Co^2+^ with apparent binding affinities of 5.6 μM for Co^2+^ and 12 μM for Ni^2+^(Furukawa et al., 2015).

### Fluorescence Imaging of *E. coli*

**FIGURE 5:**
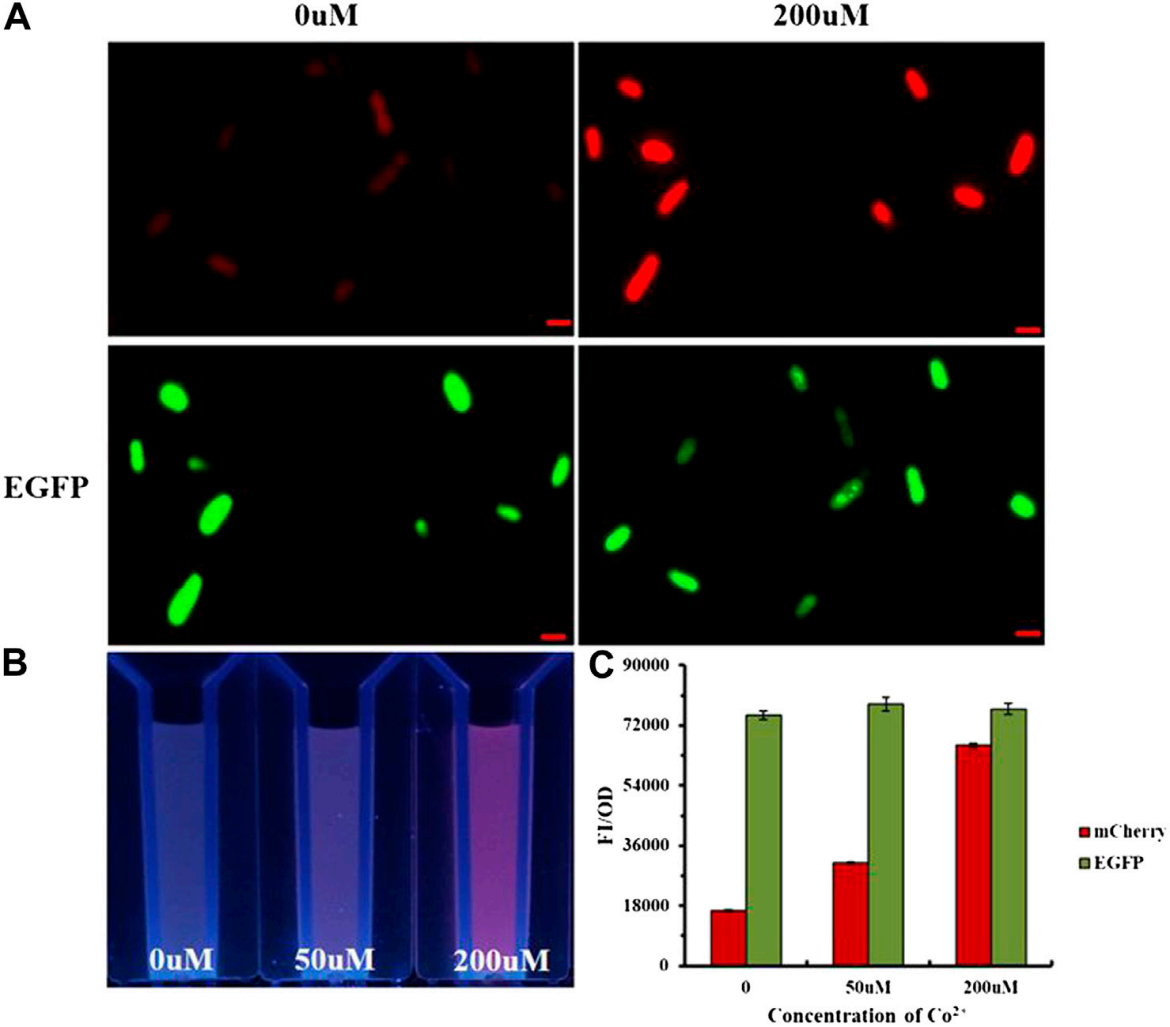
**(A)** *E.coli* imaging with Ribo2 and control under 0 μM and 200 μM. **(B)** UV photograph of *E. coli* containing Ribo2 and control under 0, 50, and 200 μM. **(C)** Concentration gradient of Co^2+^ and fluorescent measurement of **(B)**.

The imaging experiment of *E. coli* detected Co^2+^ inside the bacteria (Supplementary Figure S3). As shown in Figure 5A, pSB1C3-pCons-RBS-EGFP as the control was not affected by other factors and could express GFP steadily; the red fluorescence intensity increased with the concentration at 0–200 μM, thereby demonstrating that the metal ion sensing element for the whole-cell probe was NiCo riboswitch. The variation of fluorescence intensity was visible under UV (Figure 5B). Figure 5C also proved that the red fluorescence intensity increased with ion concentration, while the green fluorescence intensity remained unchanged.

### Riboswitch-Based Sensors can Be Used to Monitor Co^2+^/Ni^2+^ Transport

**FIGURE 6:**
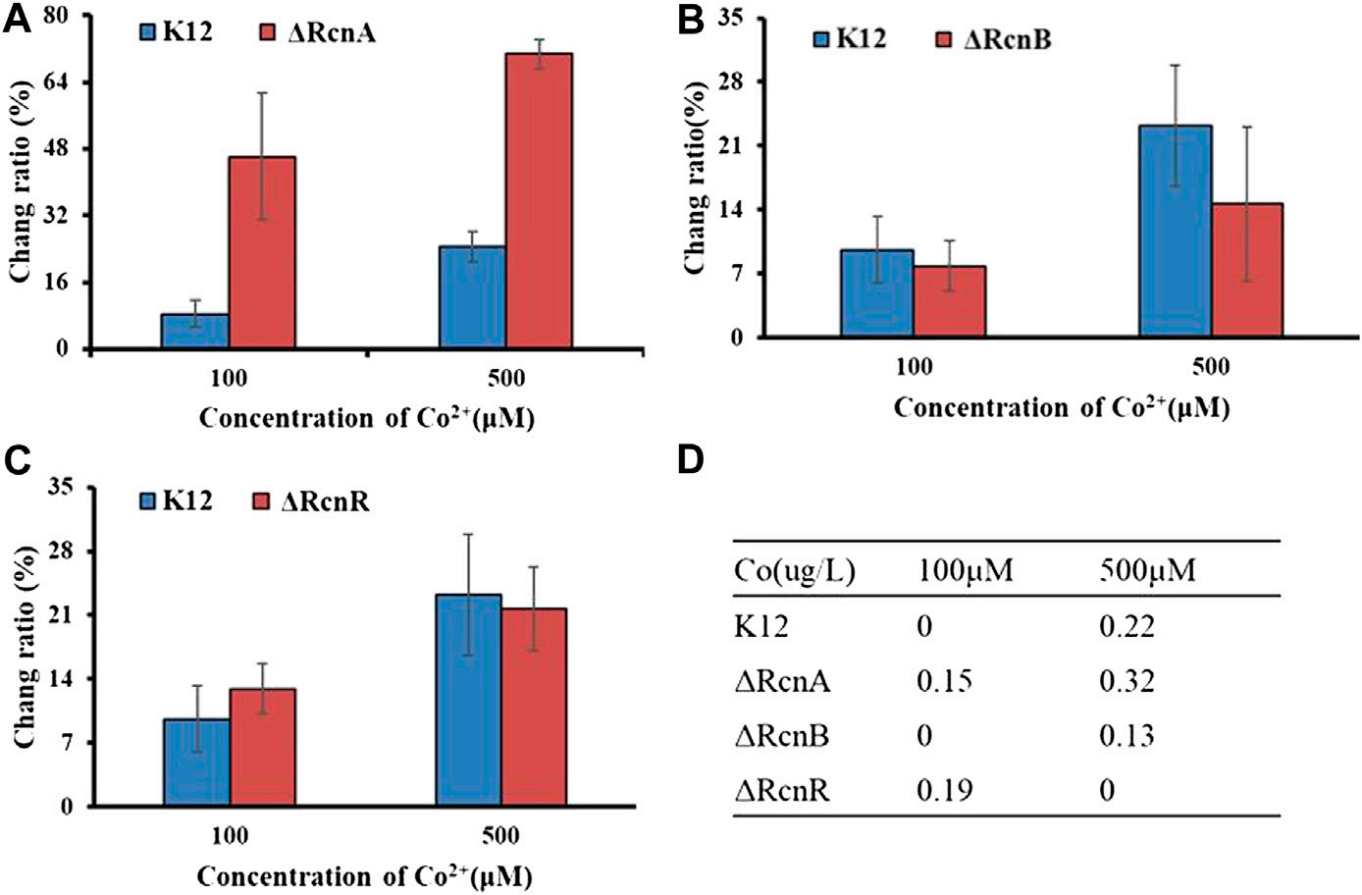
Application of Ribo2 in mutant strain: **(A)**–**(C)** Fluorescent measurement of Ribo2 in K12ΔRcnA, K12ΔRcnB, and K12ΔRcnR. **(D)** Co^2+^ concentration detected by ICP-MS.

**FIGURE 7:**
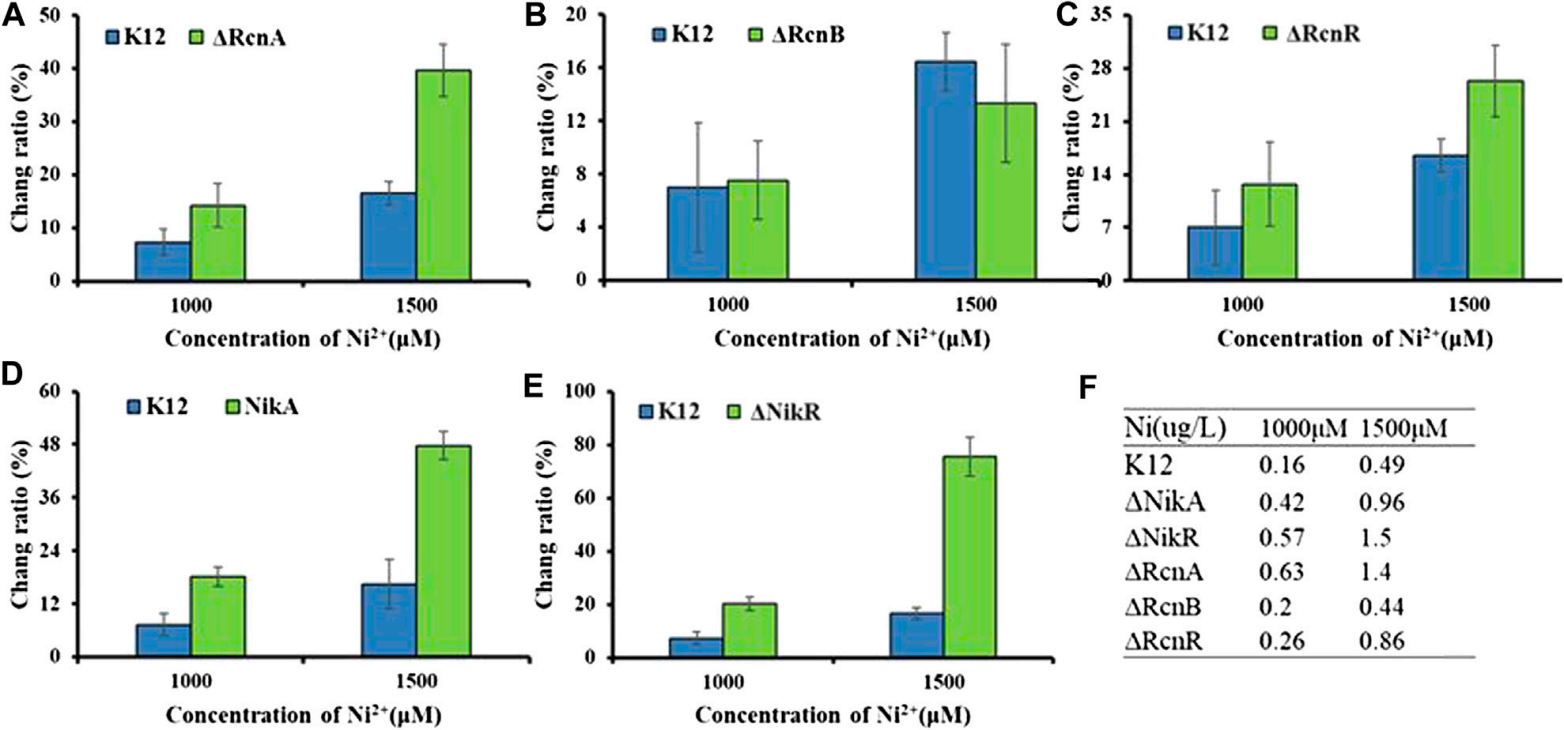
Application of Ribo2 in mutant strain: **(A)**–**(E)** Fluorescent measurement of Ribo2 in K12ΔRcnA, K12ΔRcnB, K12ΔRcnR, K12ΔNikA, and K12ΔNikR. **(D)** Ni^2+^ concentration detected by ICP-MS.

As with other metal ions, Ni^2+^ and Co^2+^ are toxic in excess. Thus, even organisms that do not have a nutritional requirement for this metal may need a mechanism to deal with excess toxicity. The common mechanism for metal resistance is metal efflux. To assess the importance of each of these genes in the transport process, (K12ΔRcnA, K12ΔRcnB, K12ΔRcnR, K12ΔNikA, and K12ΔNikR) confirmed by colony PCR (Supplementary Figure S4), we tested the effects of their deletion on intracellular Co^2+^ or Ni^2+^ levels for *E. coli* grown in media containing 100 μM and 500 μM CoCl_2_ or 1,000 μM and 1,500 μM NiCl_2_ (Figures 6, 7).

Through electro-transformation, the Ribo2 probe was transformed into the mutant strain (K12ΔRcnA, K12ΔRcnB, and K12ΔRcnR). With wild-type K12 as the control, the application of the probe in the knockout strains was investigated. Since the fluorescence intensity is related to the concentration of Ni^2+^ and Co^2+^ in bacteria, through calculating the ratio of change in fluorescence intensity relative to the blank control, the accumulation of metal ions in *E. coli* can be proved indirectly, as an attempt to understand the relevant functions of genes. According to Figure 6, the change ratio of fluorescence intensity in K12ΔRcnA knockout bacteria was significantly higher than in K12. The *rcnA* knockout resulted in accumulation of intracellular Co^2+^. Moreover, the *rcnR* knockdown caused Co^2+^ concentration increase under 100 μM, while the value in K12ΔRcnR was lower than in K12 under 500 μM. The *rcnB* knockout led to the reduction of Co^2+^ concentration. ICP-MS data revealed that the fluorescence data corresponded to ICP data, with 0 indicating below the LOD, not detected, and the reasons for “not detected” in K12ΔRcnB under 500 uM may be high concentration of metal ion leads to strong cytotoxicity. Low growth speed of K12ΔRcnR knockout bacteria and excessively low biomass are complying with the fluorescence data. Through electro-transformation, the Ribo2 probe was transformed into K12ΔRcnA, K12ΔRcnB, K12ΔRcnR, K12ΔNikA, and K12ΔNikR knockout bacteria.

As shown in Figure 7, for the concentration gradients of 1,000 μM and 1,500 μM, in wild-type and knockout strains, the fluorescence intensity was correlated with the concentration of Ni^2+^. *rcnA* and *rcnR* involved in ion efflux; detection of *rcnA* and *rcnR* caused Ni^2+^ accumulation at different levels. The change ratio of fluorescence intensity in K12ΔNikA and K12ΔNikR knockout bacteria was higher than in K12, and the value of K12ΔNikR significantly reached over that of K12ΔNikA. The ICP-MS data complied with the fluorescence data.

According to the experimental results, deletion of the respective transport gene noticeably affected Co^2+^ or Ni^2+^ efflux. The experimental data of efflux protein *rcnA* showed that upregulation of *rcnA* in *E. coli* probably promotes excess metal ions export. *rcnA* refers to one efflux pump contributing to Ni^2+^ and Co^2+^ detoxifying process in *E. coli*. *rcnA* expressing state receives the induction through Ni^2+^- and Co^2+^-based metallo-regulator *rcnR* (Blaha et al., 2011). For the mentioned reason, the efflux mechanisms show the direct regulation based on cobalt ion concentration. As opposed to the mentioned reason, uptake mechanisms show regulation through a growth substrate depending on cobalt (e.g., nitrile) or cofactor (i.e., cobalamin), instead of through the metal ion itself (Okamoto and Eltis, 2011). *nikR* refers to the best-studied nickel-dependent regulatory protein. *nikR* is a metal-responsive transcription factor that controls Ni^2+^ uptake in *E. coli* by regulating expression of a nickel-specific ATP-binding cassette (ABC) transporter (Schreiter et al., 2003). *nikA* contributes to Ni^2+^ capture and delivery to the membrane for importing in cells; it is a vital factor in the Ni^2+^ uptake (Li and Zamble 2009). *rcnB* is a periplasmic protein essential for maintaining intracellular Ni^2+^ and Co^2+^ concentrations, most likely in connection with *rcnA* (Bleriot et al., 2011). Deletion of *rcnB* conferred enhanced resistance to Ni^2+^ and Co^2+^ in *E. coli*, accompanied by decreased metal accumulating process.

## Conclusion

A main advantage of the riboswitch-based sensor is its high specificity since the highly conservative RNA sequence ensures the selectivity of the probe. As the riboswitch sensor regulates the transcription level of RNA, it is both sequential and efficient compared with the regulation on the level of the metalloprotein-based sensor which regulates the translation level of RNA. According to the results, the probe exhibited high selectivity at 1 h. The changes of intracellular Ni^2+^/Co^2+^ concentrations in *E. coli* could be detected by the riboswitch-based sensor, and the sensor could also be adopted to study Ni^2+^/Co^2+^ resistance pathways as combined with gene knockout. Since the Co^2+^/Ni^2+^uptake of pathogenic bacteria shows a relationship to pathogenicity (Neumann et al., 2017), the probes can be further applied to study on the pathogenicity of pathogenic bacteria. With the natural riboswitch, the metal riboswitch may be synthesized by artificial design, as an attempt to detect the metal resistance system in a high throughput.

## Data Availability

The raw data supporting the conclusions of this article will be made available by the authors, without undue reservation.
