# Supplementary material for: Using a Riboswitch Sensor to Detect Co2+/Ni2+ Transport in E. coli
Source: Front Chem. 2021 Feb 15;9:631909. doi: 10.3389/fchem.2021.631909 (PMC7917058; doi:10.3389/fchem.2021.631909)
Supplement: Supplementary file 1 [file datasheet1.pdf]

Supplementary Material

Construction of the whole-Cell Sensor

As shown in the **Supplementary Figure 1**, the pSB1A2 was used as the basic backbone for recombinant plasmid construction. Terminator gene (*Ter*) was amplified by PCR and digested using *XbaI*, *SpeI* and ligated into pSB1A2 between *XbaI* and *SpeI*. After confirmed by sequencing, the resulting plasmid was named pSB1A2-Ter. The gene encoding mCherry was cloned into pSBC2A-Ter by Biobrick, *mCherry* was amplified with a forward primer that contained two restriction sites *EcoRI*, *XbaI* and a reverse primer that contained a *SpeI* restriction site, the fragment was digested with *EcoRI* and *SpeI* and ligated into pSB1A2-Ter between *EcoRI* and *XbaI*. The resulting plasmid was named pSB1A2-mCherry-Ter. All NiCo riboswitch (CoNi riboswitch used here are given in **Supplementary Table 1**) fragments containing conservative promoter pCons and RBS were synthesized by GenScript. Then pCons-NiCo riboswitch-RBS was cloned into pSB1A2-mCherry-Ter by Biobrick by following the same steps mentioned above. The resulting plasmid was named pSB1A2-pCons-NiCo riboswitch-RBS-mCherry-Ter.

All restriction enzymes described above used QuickCut restriction enzymes from TaKaRa. All ligations were performed using T4 DNA ligase from Fermentas. PCR reactions were performed using Taq DNA Polymerase (TaKaRa). Reactions were carried out essentially according to supplier. All plasmidswere confirmed by sequencing (GenScript).

Supplementary Table 1 CoNi riboswitch source and detailed sequence

**Gene name**	**Gene source**	**Sequence**
Ribo1	*Clostridium botulinum*	GGGGTACAAACTGATCAGGCCGATAAATTATTTGATTTATGGAGCCGGGCCATTTTTGTGGCAACAGGATATTTAATACCTGTGGGACAGTATAT
Ribo2	*Erysipelotrichaceae bacterium*	GGGGTACAAACTGAGCAGGCAAATGACCAGAGCGGTCATGCAGCCGGGCTGCGAAAGCGGCAACAGATGATTACACGCACATCTGTGGGACAGTTGTATATTCCACAGATGTTTTTT
Ribo3	*Clostridium cellulolyticum*	GGGAGTACAAACTGAGCAGGCGATGGACCTTTCATAGAGGTACATGGGGCCGGGCCACCCAGTGAGTGGCAGCAGATTGCAATCATGCACATCTGTGGGACAGTAGTATGTTCCACGGGTGTGCTTTTTTT

Construction of mutant strain

Genes were deleted by the insertion of Kan^r cassettes using the λ Red-recombinase system refer to a previously published protocol(Datsenko and Wanner 2000). The recombinant plasmid pUC57-Kan containing Kan^r gene and homologous sequences of target genes was constructed (As shown in the **Supplementary Figure 2**). A linearized DNA flanked by homologous sequences for gene deletion was obtained by PCR, PCR products were purified, digested with *DpnI*, repurified and suspended in ddH_2O and then transformed into *Escherichia coli* K12 strain containing plasmid pKD46. Catalyzed by λ Red-recombinase system, target gene was knocked out while Kan^r was knocked in. Electroporation-competent cells containing plasmid pKD46 were prepared. Electroporation of linear homologous fragment was done by using a MicroPulser (BioRad), 1.8kV, 1 pulse, 0.1 cm cuvette. Shocked cells were added to 1 ml SOC, incubated 1 h at 37 ℃, the products were coated onto agar to select kanamycin transformant and confirmed by PCR (As shown in the **Supplementary Figure 3**). pKD46 was a temperature-sensitive plasmid, which was lost automatically when the culture condition was higher than 42 ℃.

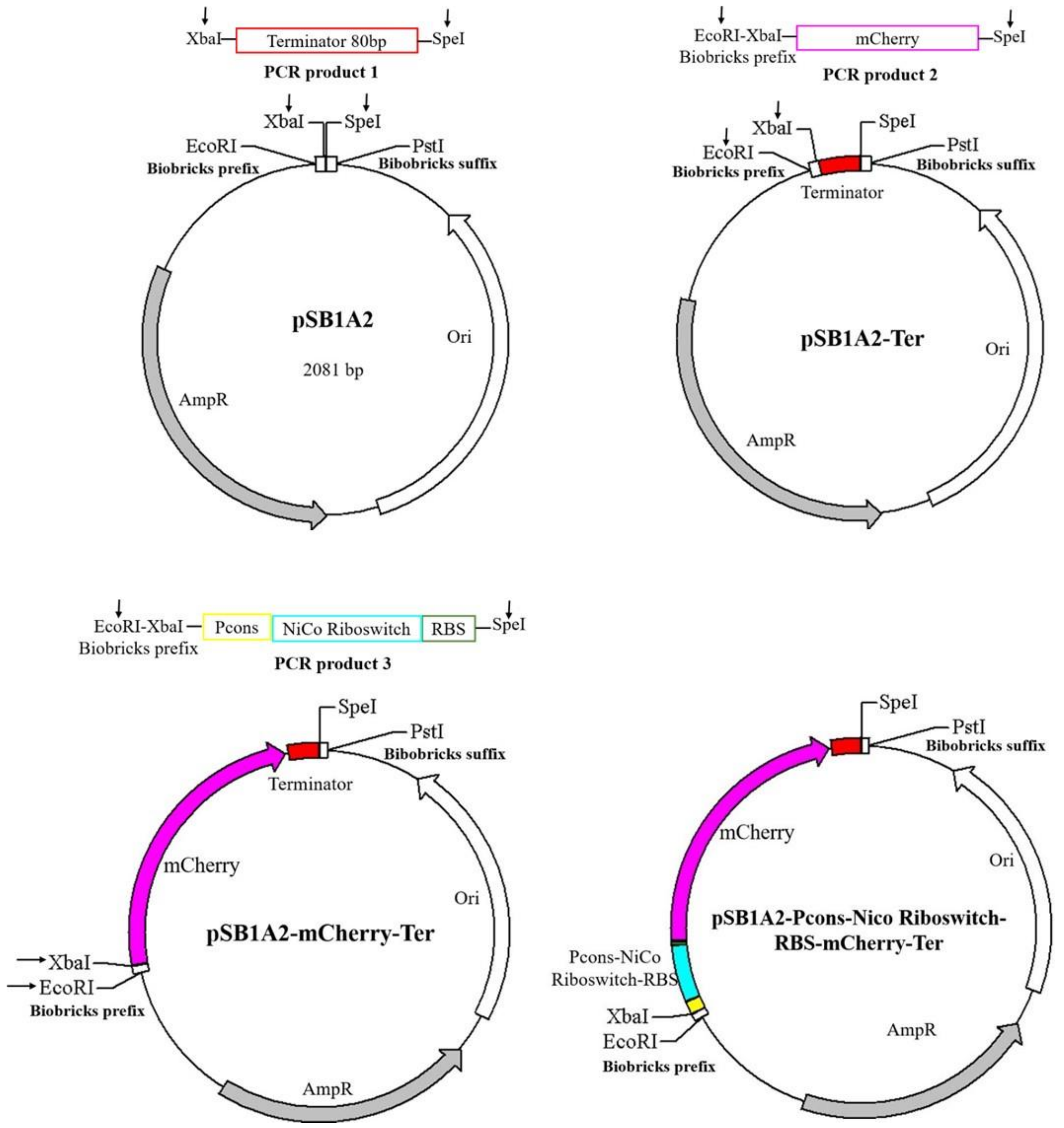

Supplementary Figure 2. Cloning scheme for NiCo riboswitch sensor constructs showing the relevant plasmid features.

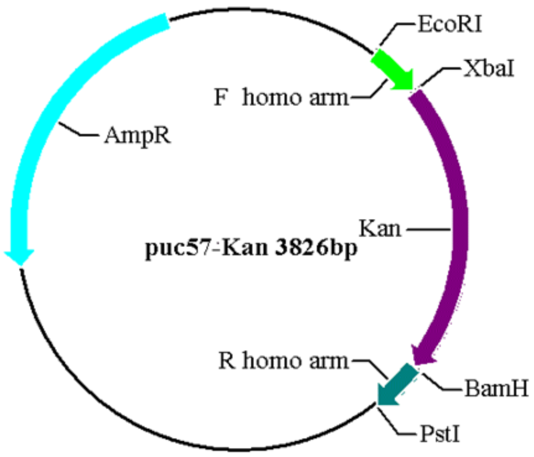

Supplementary Figure 2. Clone scheme for pUC57-Kan.

48

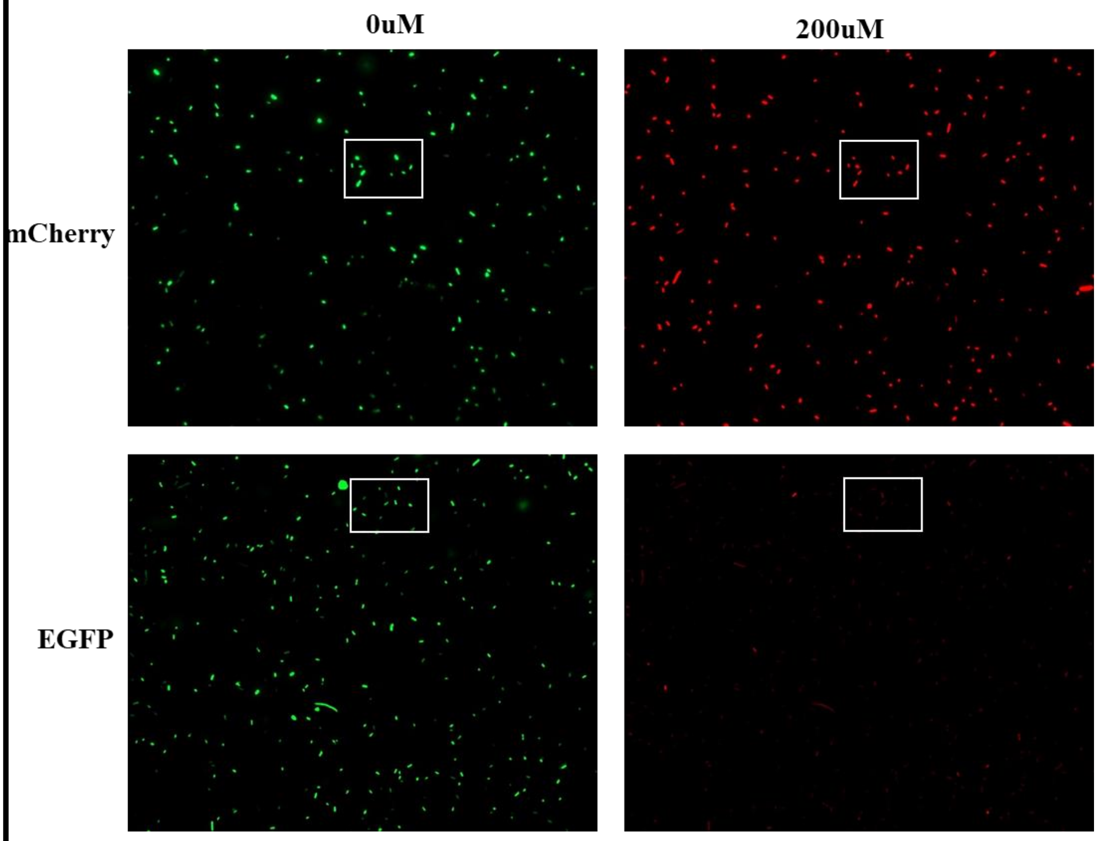

Supplementary Figure 3. *E.coli* imaging with Ribo2 and control under 0 μM and 200 μM. The enlarged part of Figure 5A is marked by the box.

49

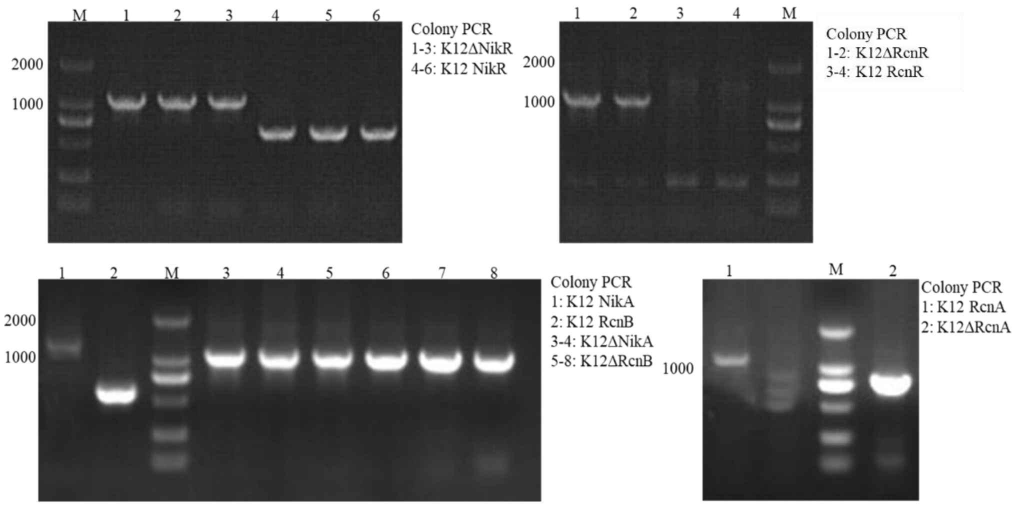

Supplementary Figure 4. K12ΔRcnA， K12ΔRcnB, K12ΔRcnR, K12ΔNikA, K12ΔNikR confirmed by colony PCR.

REFERENCES

Datsenko, K. A., and B. L. Wanner. 2000. "One-step inactivation of chromosomal genes in Escherichia coli K-12 using PCR products." *Proceedings of the National Academy of Sciences of the United States of America* no. 97 (12):6640-6645. doi: 10.1073/pnas.120163297.
