# Supplementary material for: Using a Riboswitch Sensor to Detect Co2+/Ni2+ Transport in E. coli
Source: Front Chem. 2021 Feb 15;9:631909. doi: 10.3389/fchem.2021.631909 (PMC7917058; doi:10.3389/fchem.2021.631909)
Supplement: Supplementary file 2 [file table1.docx]

***Supplementary Material***

**Construction of the whole-Cell Sensor**

As shown in the **Supplementary Figure 1**, the pSB1A2 was used as the basic backbone for recombinant plasmid construction. Terminator gene (*Ter*) was amplified by PCR and digested using *XbaI*, *SpeI* and ligated into pSB1A2 between *XbaI* and *SpeI*. After confirmed by sequencing, the resulting plasmid was named pSB1A2-Ter. The gene encoding mCherry was cloned into pSBC2A-Ter by Biobrick, *mCherry* was amplified with a forward primer that contained two restriction sites *EcoRI*, *XbaI* and a reverse primer that contained a *SpeI* restriction site, the fragment was digested with *EcoRI* and *SpeI* and ligated into pSB1A2-Ter between *EcoRI* and *XbaI*. The resulting plasmid was named pSB1A2-mCherry-Ter. All NiCo riboswitch (CoNi riboswitch used here are given in **Supplementary Table 1**) fragments containing conservative promoter pCons and RBS were synthesized by GenScript. Then pCons-NiCo riboswitch-RBS was cloned into pSB1A2-mCherry-Ter by Biobrick by following the same steps mentioned above. The resulting plasmid was named pSB1A2-pCons-NiCo riboswitch-RBS-mCherry-Ter.

All restriction digests described above used QuickCut restriction enzymes from TaKaRa. All ligations were performed using T4 DNA ligase from Fermentas. PCR reactions were performed using Taq DNA Polymerase (TaKaRa). Reactions were carried out essentially according to supplier. All plasmid constructs were confirmed by sequencing (GenScript).

**Supplementary Table 1** CoNi riboswitch source and detailed sequence

| **Gene name** | **Gene source** | **Sequence** |
| --- | --- | --- |
| **Ribo 1** | ***Clostridium***  ***botulinum*** | GGGGTACAAACTGATCAGGCCGATAAATTATTTGATTTATGGAGCCGGGCCATTTTTGTGGCAACAGGATATTTAATACCTGTGGGACAGTATAT |
| **Ribo 2** | ***Erysipelotrichaceae***  ***bacterium*** | GGGGTACAAACTGAGCAGGCAAATGACCAGAGCGGTCATGCAGCCGGGCTGCGAAAGCGGCAACAGATGATTACACGCACATCTGTGGGACAGTTGTATATTCCACAGATGTTTTTT |
| **Ribo3** | ***Clostridium cellulolyticu****m* | GGGAGTACAAACTGAGCAGGCGATGGACCTTTCATAGAGGTACATGGGGCCGGGCCACCCAGTGAGTGGCAGCAGATTGCAATCATGCACATCTGTGGGACAGTAGTATGTTCCACGGGTGTGCTTTTTT |

**Construction of mutant strain**

Genes were deleted by the insertion of Kan^r^ cassettes using the λRed-recombinase system refer to a previously published protocol([Datsenko and Wanner 2000](#_ENREF_1)). The recombinant plasmid pUC57-Kan containing Kan^r^ gene and homologous sequences of target genes was constructed (As shown in the **Supplementary Figure 2**). A linearized DNA flanked by homologous sequences for gene deletion was obtained by PCR , PCR products were gel-purified, digested with *DpnI*, repurified and suspended in ddH_2_O and then transformed into a *Escherichia coli* K12 strain containing plasmid pKD46. Catalyzed byλRed-recombinase system, target gene was knocked out while Kan^r^ was knocked in. Electroporation-competent cells containing plasmid pKD46 were prepared. Electroporation of linear homologous fragment was done by using a MicroPulser (BioRad), 1.8kV, 1 pulse, 0.1cm cuvette. Shocked cells were added to 1ml SOC, incubated 1h at 37°C, the products were coated onto agar to select kanamycin transformant and confirmed by PCR (As shown in the **Supplementary Figure 3**). After primary selection, mutants were colony purified once at 37°C and then tested for ampicillin sensitivity to test for loss of the pKD46. If it was not lost, then a few were colony-purified once at 42°C and similarly tested.

**Supplementary Figure 1.** Cloning scheme for NiCo riboswitch sensor constructs showing the relevant plasmid features.


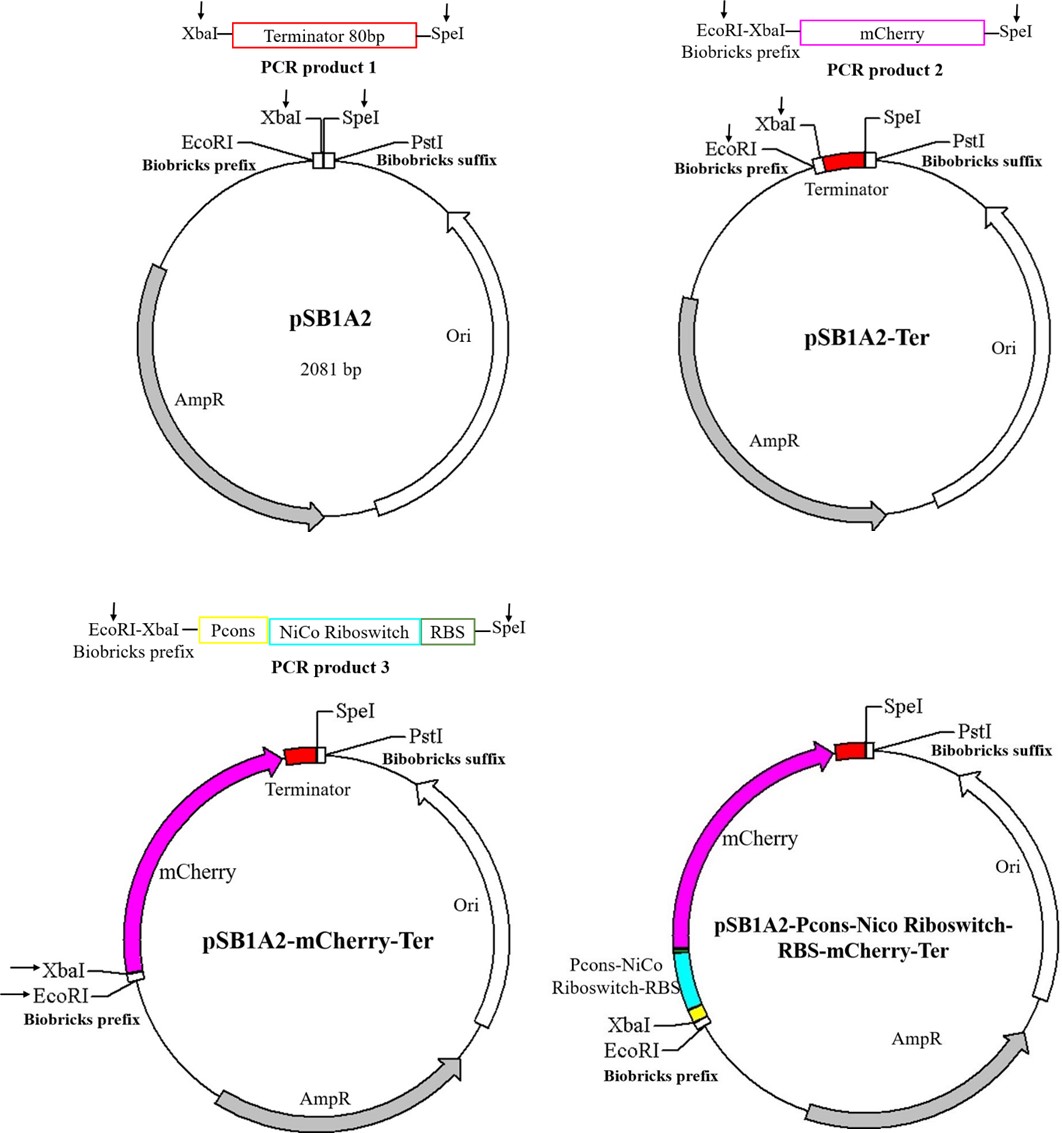

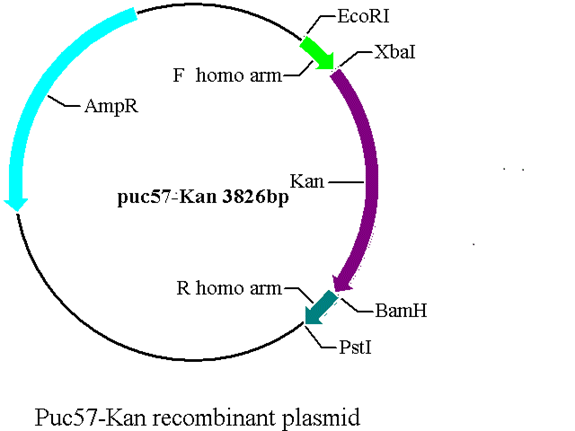


**Supplementary Figure 2.** Clone scheme for pUC57-Kan

**Supplementary Figure 3.** *E.coli* imaging with Ribo2 and control under 0μM and 200μM. The enlarged part of Figure 5A is marked by the box.


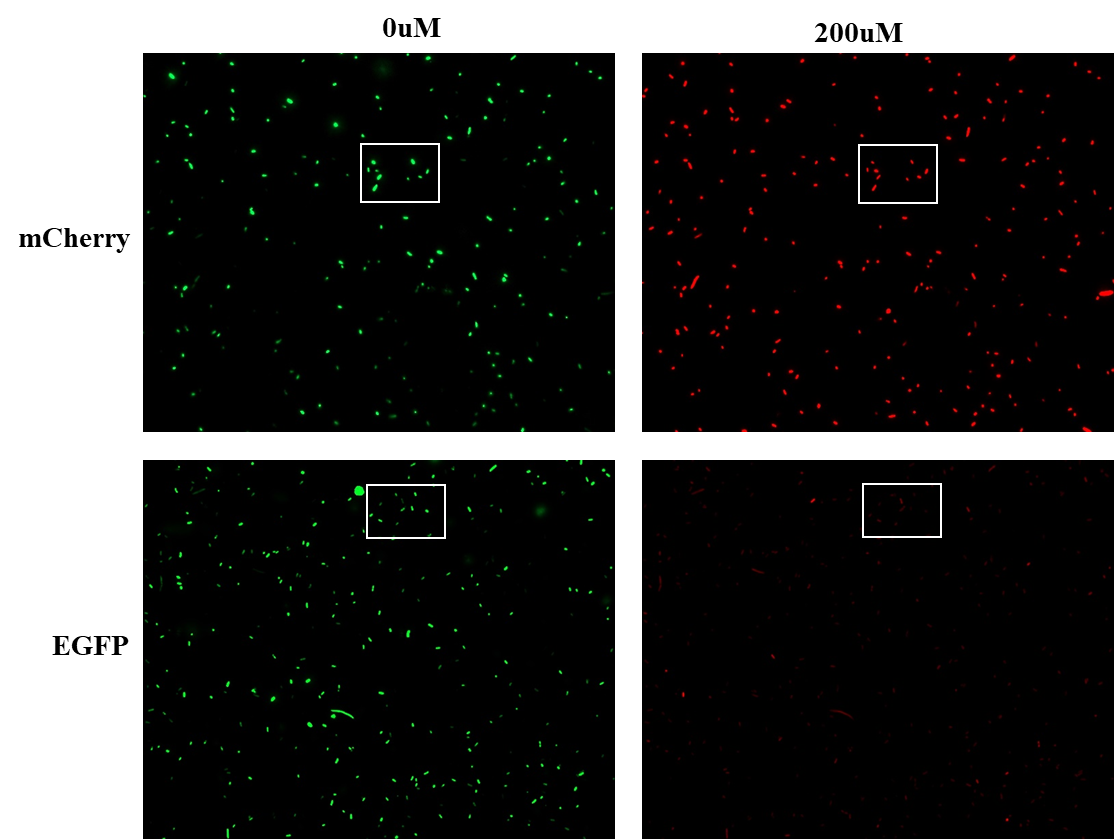


**REFERENCES**

**Supplementary Figure 4.** K12ΔRcnA，K12ΔRcnB, K12ΔRcnR, K12ΔNikA, K12ΔNikR confirmed by colony PCR.


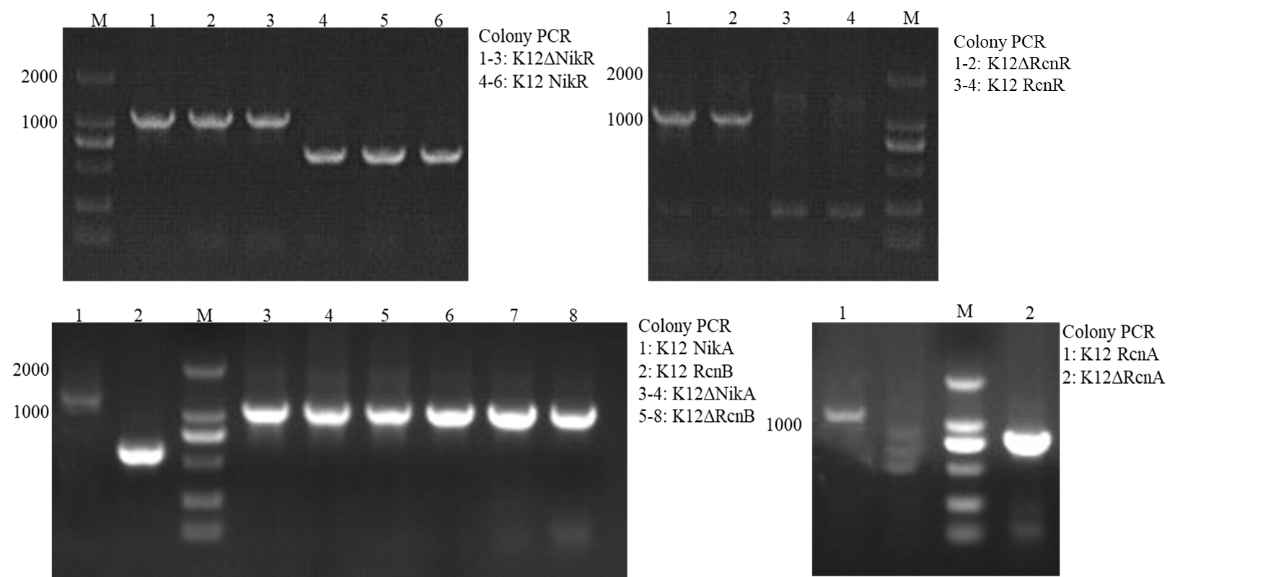


Datsenko, K. A., and B. L. Wanner. 2000. "One-step inactivation of chromosomal genes in Escherichia coli K-12 using PCR products." *Proceedings of the National Academy of Sciences of the United States of America* no. 97 (12):6640-6645. doi: 10.1073/pnas.120163297.
